# Diagonal ear lobe crease and atherosclerosis: A review of the medical literature and dental implications

**DOI:** 10.4317/medoral.17390

**Published:** 2011-07-15

**Authors:** Arthur H Friedlander, José López-López, Eugenio Velasco-Ortega

**Affiliations:** 1 DMD, Associate Chief of Staff and Director of Graduate Medical Education, VA Greater Los Angeles Healthcare System. Director of Quality Assurance, Hospital Dental Service, UCLA Medical Center. Professor of Oral and Maxillofacial Surgery, School of Dentistry, University of California Los Angeles, CA; 2 MD, PhD, Medical Doctor Specialist in Stomatology. Chair Post-Grade of Oral Medicine and Post-Grade of Dentistry in Oncologics and patients with immunodeficiencies. University of Barcelona, Catalonia, Spain. Professor of Department of Stomatology, School of Dentistry, University of Barcelona, Catalonia, Spain; 3 MD, PhD, Medical Doctor Specialist in Stomatology. Chair Post-Grade of Oral Implantology, University of Sevilla, Andalusia, Spain. Professor of Department of Stomatology, School of Dentistry, University of Sevilla, Andalusia, Spain

## Abstract

In Spain a significant number of individuals die from atherosclerotic disease of the coronary and carotid arteries without having classic risk factors and prodromal symptoms. The diagonal ear lobe crease (DELC) has been characterized in the medical literature as a surrogate marker which can identify high risk patients having occult atherosclerosis. This topic however has not been examined in either the medical or dental literature emanating from Spain. The majority of clinical, angiography and postmortem reports support the premise that DELC is a valuable extravascular physical sign able to distinguish some patients at risk of succumbing to atherosclerosis of the coronary arteries. A minority of studies have however failed to support this hypothesis. More recently reports using B mode ultrasound have also linked DELC to atherosclerosis of the carotid artery and another report has related DELC to the presence of calcified carotid artery atheromas on panoramic radiographs. DELC is readily visible during head and neck cancer screening examinations. In conjunction with the patient’s medical history, vital signs, and panoramic radiograph, the DELC may assist in atherosclerotic risk assessment.

** Key words:** Diagonal ear lobe crease, atherosclerosis disease, calcified carotid artery, atheromas, panoramic radiographs.

## Introduction

Atherosclerosis is a major source of disability and death in Spain. In 2002, coronary artery atherosclerosis (CAA) resulted in 45,000 Spanish citizens suffering a fatal myocardial infarction and carotid artery atherosclerosis resulted in the stroke associated death of an additional 34,000 persons (1). Many of these adverse events were in individuals free of prodromal symptoms and often without classical risk factors such as hypertension, hyperlipidemia, diabetes, tobacco use, obesity, or sedentary a lifestyle. This “detection gap” has spurred clinicians to attempt to find extravascular physical signs which might identify individuals at high risk of atherosclerosis. Some extravascular physical signs may present in the maxillofacial region and be visible to the dentist: obvious examples include arcus senilis, xanthelasma and male pattern baldness. But the specificity and, sensitivity of these stigmata, and their independence from confounding variables have been questioned (2,3).

**Figure 1:**
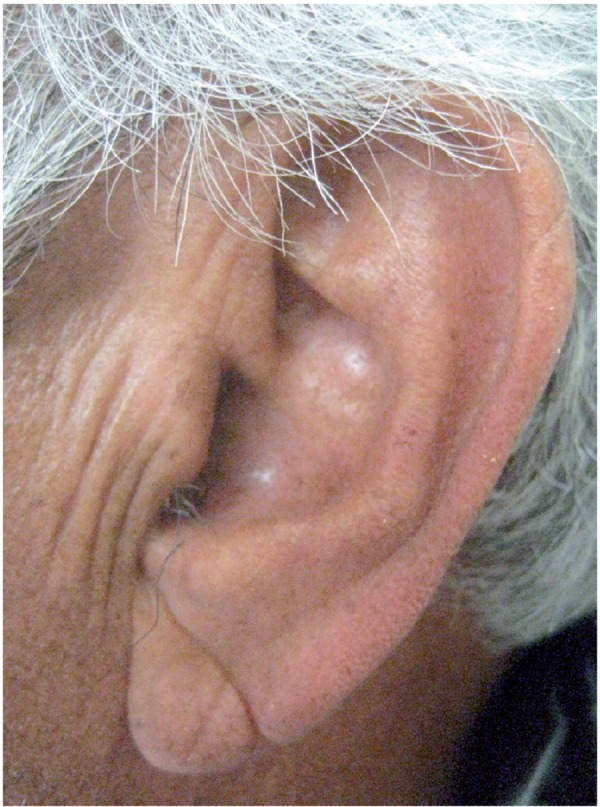
A 69 year old man with a very well defined diagonal ear lobe crease on the left side.

**Figure 2:**
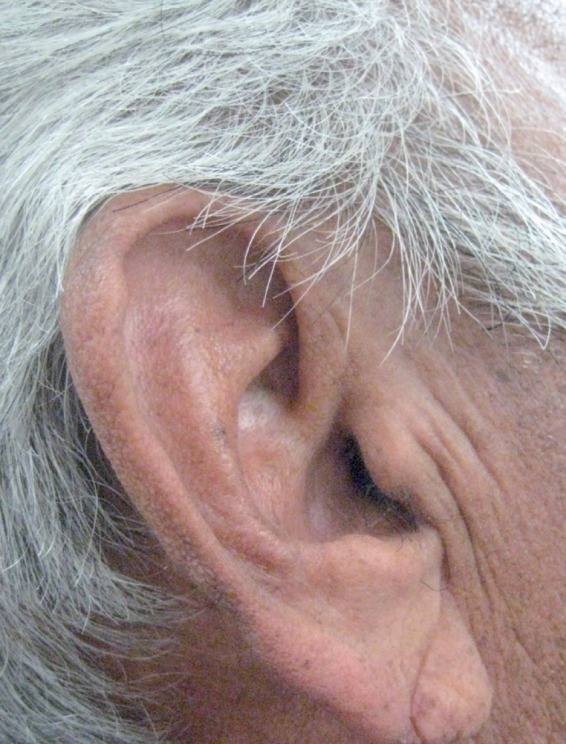
The same patient with a somewhat less distinct diagonal ear lobe crease on the right side.

Preliminary uncontrolled observations by a pulmonologist that a “positive ear-lobe sign” (similar to the anterior-posterior crease depicted in Figs. 1,2), is associated with the development of premature atherosclerosis of the coronary arteries were heralded in a communication as long ago as 1973 (4). In a “Letter to the Editor” of the New England Journal of Medicine, Sanders T. Frank, M.D. described a group of 20 patients, from his practice, near Los Angeles, California USA who were 60 years of age or younger and who had. In addition to the ear-lobe sign (which was usually bilateral) angina. The electrocardiogram and angiography confirmed ischemic changes and CAA (4). The prognostic significance of “Frank’s sign” now termed “diagonal ear-lobe crease” (DELC) has subsequently been substantiated in more than 40 separate studies, a number of which will be described in this review. Prior to Frank’s description, aficionados of Roman sculpture might have seen but not grasped the significance of busts portraying the emperor Hadrian prominently displayed bilateral ear lobe creases (5). Adrian, born in Italica, 10 km from present day Sevilla, is believed to have died from congestive heart failure secondary to uncontrolled hypertension and CAA. Roman portrait sculpture is considered to be highly accurate and detailed, sculptures of other Roman emperors and nobles in museums housing Hadrian’s bursts do not have DELC.

## Evidence Supporting Relationship between DELC and Coronary Artery Atherosclerosis

Frank’s report was quickly followed in 1974 by the publication of the first controlled clinical study in which DELC (unilateral or bilateral) was shown to be significantly more common (47%) among all age groups of patients (N=531) hospitalized in a USA coronary-care unit after an acute myocardial infarction than among age-matched control subjects (30%) with no clinical evidence of CAA (6).

In 1978, Kaukola, an internist at University Hospital in Helsinki, Finland reported on the correlation between DELC and CAA (7). He evaluated 219 patients (165 men, and 54 women, age range 32-65 years). Who evidenced CAA by virtue of admission to hospital with an acute myocardial infarction for the presence of DELC. He reported that 69% of the men and 69% of the women had a DELC. For comparison, Kaukola evaluated a like-aged control group consisting of 290 subjects (236 men and 54 women) who attended an employee health clinic. DELC was present in 24% of the control group and specifically in 28% of the men and 9% of the women. The prevalence rate of DELC was significantly greater among the patients sustaining an acute myocardial infarction than among the controls.

The same author, in a second paper, published as a Letter to the Editor in the New England Journal of Medicine evaluated 286 randomly selected patients (age range 26-66) requiring coronary angiography at Helsinki University Central Hospital because of typical signs of CAA, that is angina pectoris and ischemic electrocardiographic changes (8). Two-hundred individuals (174 men and 26 women, mean age 48) had at least 50% stenosis (CAA) in one or more of the epicardial arteries (left main coronary artery, left anterior descending coronary artery, left circumflex coronary artery, and right coronary artery). Eighty six individuals (35 men, 51 women, mean age 51) were with negligible/low degree stenosis (≤50%; no CAA). Of the 200 individuals with CAA, 72% had DELC, whereas of the 86 individuals without significant stenosis, only 21% had DELC. The difference in DELC prevalence rates between the groups was highly significant. Furthermore, the prevalence of DELC increased with severity of CAA, being positive in 52% of patients with single vessel disease, and 79% of patients with double or triple vessel disease.

A group of physicians from Copenhagen, Denmark examined all patients (203 women, 320 men) admitted to a medical-surgical unit during a two month period of time for the presence of DELC (9). In patients between the ages of 50 and 59 the prevalence rate (46.8%) of DELC in those with an acute myocardial infarction was significantly greater than in an age matched control group (31.6%). Approximately 15 years later, again in Denmark, the Copenhagen City Heart Study, prospectively analyzed a healthy population (N = 14,223) of outpatients to determine the relationship between right-sided DELC and the development of a first acute myocardial infarction (AMI) as an indicator of CAA (10). All enrollees were followed for a 6.5 year interval and those with the DELC were noted to have a statistically significant 1.4-fold (the 95% confidence interval for relative risk was 1.1 – 1.7) increased risk of developing an AMI after controlling for age and sex. 

A Turkish study of 415 men and women (mean age 58.9 years) requiring coronary artery angiography for stable angina with a positive stress test, unstable angina, or history of prior myocardial infarct were also evaluated for bilateral DELC (11). There was a highly significant statistically greater prevalence of DELC (51.4%) in those patients with a positive angiogram (as defined as > 70% stenosis of the luminal diameter in 1 or more of the three epicardial arteries) than in those whose angiogram was normal (15.1%). The observed sensitivity of the bilateral DELC for the diagnosis of CAA was 51%, the specificity 85%, the positive predictive value 89%, and negative predictive value 41%. In a similar Japanese angiography study two-hundred patients were divided into two groups based upon the results of imaging (12). One-hundred and nineteen patients had greater than 50% luminal narrowing of at least one major coronary artery and 81 had no significant atherosclerotic changes in their vessels. DELC was present in 26.1% with stenosis but in only 3.7% without stenosis. The differences between the groups were statistically significant.

DELC has also been shown to have predictive significance. In one prospective clinical study, of newly admitted hospitalized patients (N = 108), matched for sex, race, and age and having at least one DELC but free of CAA followed for an 8-10 year period. Cardiac event rates (cardiac death, nonfatal myocardial infarction, need for coronary artery by-pass surgery) for those with DELC were higher (10.4 events per 100 patient-years) than for those without DELC (1.4 events per 100 patient-years) (13). The difference between groups was statistically significant. The cardiac death rates (due to acute myocardial infarction or sudden death) were likewise higher for patients with DELC (8.0 per 100 patient years) than for those without DELC (0.9 per 100 patient-years). The difference between groups was significant. A postmortem study in UK evaluated the presence of well-defined bilateral DELC and CAA among a group of men (N = 206) and women (N = 161) with a mean age of approximately 74 years (14). The relative risk of a male (mean age 70.4, ±10.9) with well-defined bilateral DELC having severe coronary artery atherosclerosis defined as 75% stenosis was 1.64 and for a female (mean age 77.4 ±10.5) it was 3.65. Furthermore, CAA identified as the cause of death was nearly twice as likely in men with well-defined bilateral DELC and 3.42 times in women with similar stigmata. The sensitivity of well-defined bilateral DELC for detecting severe CAA was 62.1% for men and 69.2% for women. The specificity was 65.9% in men and 78.0% in women.

Similarly, a British forensic necropsy study convened to determine the medical/legal cause of death of unselected consecutive adult cases (N = 303) demonstrated that the proportion of DELC in those aged ≥ 55 was significantly higher than in younger subjects (74% vs. 39%) (15). The proportion of DELC in males and females was similar (72%) vs. (67%). Men with DELC (unilateral or bilateral) had a risk of cardiovascular cause of death of 1.55 times higher than those without a crease. Women with a DELC (unilateral or bilateral) had a risk of cardiovascular cause of death of 1.74 times higher than those without a crease.

A Swedish forensic necropsy study of the death of 420 males and 100 females (mean age 56) residing in Linkoping, Sweden (16), demonstrated that 55% had DELC. The crease was equally common in both sexes (53.8% of men and 60% of women; the differences between them not being statistically significant). A unilateral DELC was noted in 8.8% of individuals whereas a bilateral DELC was noted in 91.2% of individuals. Among participants less than 40 years of age (N = 103) 19% had a DELC, of those 40-59 years (N = 199) 50% had a DELC and for those over age 60 (N = 218), 75% had a DELC. The study also showed that in both sexes, DELC was strongly predictive of both the presence and degree of CAA in the left anterior descending, circumflex, and right coronary arteries. The sensitivity, specificity and positive and negative predictive values for those less than age 40 were 0.68, 0.84, 0.80, 0.72; for those between ages 40-50 years they were 0.68, 0.60, 0.63, 0.66, and for those over age 60 they were 0.79, 0.33, 0.51, 0.61. Furthermore, the study also revealed that DELC was strongly associated with sudden cardiac death in men, but not women, and in both sexes with generalized atherosclerosis as evidenced by changes in the aorta and cerebral vessels.

There are numerous other studies which have demonstrated the very strong relationship between DELC and CAA, however there are confounding issues related to DELC such as its prevalence in different ethnic groups as well as the effect of known atherogenic risk factors. For example, as noted earlier, among northern Europeans coming to autopsy (mean age 60) the prevalence of DELC is 75%,17 far greater than the prevalence among native Japanese where it is only 5% (12). In San Francisco, California USA, a study was conducted using police arrest photographs (“Mug shots”) to determine the prevalence of DELC in various racial groups of persons aged 46-65 years (17). The rates of DELC were as follows: Hawaiian-Samoans 0% (0/12), Chinese 21% (6/29), Blacks 37.9% (44/116), Latin-Americans 47.5% (19/40) and Caucasians 50.8% (62/122). Relative to specific known atherogenic risk factors, DELC has been reported to be associated with hypertension (12, 18-20), hypercholesterolemia (4,12), smoking (4,12,21) obesity (21) and diabetes mellitus (12,21,22)

## Evidence Refuting the Relationship between Coronary Artery Atherosclerosis and DELC

Over the past 35 years there have been a number of published studies which have failed to substantiate the relationship between DELC and CAA (23,24). A number of additional but more substantial studies as well as a comprehensive letter to the editor are reviewed below.

In a prospective study conducted in Ireland, researchers assessed the relationship between DELC and the severity of CAA as defined by the volume of myocardium at jeopardy of necrosis because of the anatomical location and number of stenotic lesions exhibited by 125 consecutive patients undergoing coronary angiography (25). The researchers reported that individuals with DELC were more likely to have moderate or severe disease than individuals without DELC. Whereas individuals without DELC were more likely to have normal or mild disease than individuals with DELC. However, using discriminating function analysis the authors determined that for the group of patients as a whole, DELC was not significantly related to the presence or severity of CAA.

In a prospective study conducted at the University of Massachusetts Medical Center, researchers evaluated the relationship between DELC and CAA among 261 consecutively admitted male patients (mean age, range not provided) requiring coronary artery angiography. The angiograms were classified as normal (no lesions detected), minor lesions (< 50% occlusion), and single-vessel (> 50% occlusion), double-vessel, and triple-vessel disease. CAA of varying severity was noted in 67% of individuals with DELC and 67% of individuals without DELC. There being no difference between the groups, the authors concluded that the results of their study did not support DELC as a marker of CAA (26).

In the early 1990’s Kuon et al., prospectively evaluated 670 consecutive patients (information relative to age and sex distribution not provided) requiring coronary angiography at an acute care hospital in Erlangen, Germany (27). They determined that the presence of DELC was not significantly related to CAA (as defined by one or more coronary artery vessels having > 70% stenosis) because 55.9% of patients with DELC and 55% of patients without DELC had vessel disease; the rates being almost identical.

In 2009 in a letter to the editor in the American Journal of Forensic Medicine and Pathology, Koracevic and Atanaskovic reported on their previously unpublished study which they had conducted 13 years earlier (28). These investigators evaluated 78 unselected hospitalized patients in Nis, Serbia. There were 60 men and 28 women in the study with a mean age of 60.4. Without providing further detail the authors concluded that irrespective of whether patients had unilateral or bilateral DELC or if the crease was deep or superficial (< 1 mm) it was not a marker of CAA as defined by the presence or history of stable angina, unstable angina, myocardial infarction or insertion of a coronary artery by-pass graft.

## Proposed Etiology and Pathogenesis Responsible for the Relationship between DELC and ACA

In the 1970’s it was postulated that DELC and CAA arose simultaneously because the ear lobe and heart are supplied by “end arteries” without the possibility for collateral circulation. Others suggested that the generalized loss and degeneration of elastin and tears in the elastic fibers seen in biopsy specimens taken from the ear lobes of persons affected with CAA reflected the microvascular disease that was also present in the coronary bed but in a separate histologic study degenerative changes in the ear lobes of patients having sustained a fatal myocardial infarction could not be identified (7). Others postulate that DELC may reflect skin aging, since not only are DELC rare in infants (18) but skin aging parallels the aging changes in coronary arteries (29). More recently, a study of Japanese male patients having DELC and multiple risk factors (metabolic syndrome) for CAA demonstrated shortened telomeres (extreme ends of chromosomal DNA) in the subjects’ peripheral white blood cells, again implicating aging (30).

## Dental Implications

In 2007, Celik S et al., published the first report on the relationship between DELC and carotid artery atherosclerosis imaged by B-mode ultrasound among a group of apparently healthy Turkish subjects attending a medical clinic for a standard medical check-up (31). This author measured carotid artery intima-media thickness (CIMT), a widely used surrogate marker for localized and systemic atherosclerotic disease, and an independent prognostic indicator of future adverse cardiovascular events. Subjects with DELC (unilateral or bilateral) had significantly higher CIMT compared to age and gender matched controls without DELC.

In 2009, Shrestha I et al., evaluated 212 patients (106 males, 106 females with a mean age of 67) in Japan requiring a B-mode ultrasound evaluation of the extracranial carotid arteries because of a prior history of cardiovascular/cerebrovascular disease or for pre-operative screening purposes (32). Approximately 30% of the patients had a DELC which demonstrated an independent association with CIMT in multivariate regression analysis after adjusting for age, sex and hypertension. There was also a significant correlation between DELC and atherosclerotic plaque (defined as CIMT of ≥ 1.1 mm) number (total number of plaques) and plaque score (sum of the heights of all plaques). The relationship between DELC and plaque may be even more meaningful than the relationship between DELC and CIMT given that some believe that the presence of carotid plaque is more closely associated with extensive coronary artery atherosclerosis than is CIMT (33).

**Figure 3:**
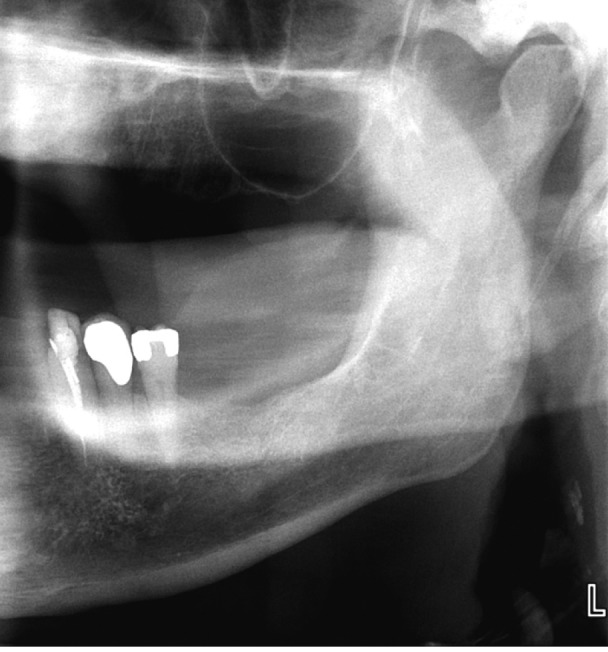
A cropped panoramic radiograph of the same individual in Figures 1 and 2. Note the two calcified carotid artery atheroma superior to the prominent semi lunar shaped epiglottis and the letter “L” marker.

In 2010, in a Letter to the Editor of the Journal of Oral and Maxillofacial Surgery, a dental researcher prompted by Celik’s report (Shrestha’s manuscript was still in-press), reported on the results of an observational study on the prevalence of DELC in 10 consecutive neurologically asymptomatic patients (mean age 65, range 61-74) referred for confirmation of the presence of a calcified carotid artery atheroma (CCAA) on their dental radiographs (Fig. 3) (34). The researcher determined that all 10 radiographs showed CCAA (7 bilateral and 3 unilateral) and these findings were further substantiated by B-mode ultrasound studies. The patients were then examined in a dental chair positioned at an angle of 45˚ to avoid artifact (26). DELC was considered present if there was a unilateral or bilateral downwards (anterior-posterior) oblique (at approximately 45˚) wrinkle in the ear lobe that extended for a distance of 80% or more of the lobe length (35). On clinical examination, 8 patients had bilateral DELC and one patient had a unilateral crease. 

The results of the above noted pilot study are important even though it was of small sample size and uncontrolled given that a team of dentists and neurologists, in an earlier controlled study (when the importance of DELC was unrecognized), followed a group (N = 46) of patients (mean age 68) with CCAA on their panoramic radiographs for an average follow-up period of 3.6 years (36). 

During this time, coronary artery and carotid artery atherosclerosis as evidenced by adverse vascular events such as angina requiring hospitalization, need for coronary artery revascularization surgery, non-fatal myocardial infarct, stroke, and transient ischemic attack were significantly more common among the study patients than among controls. These results demonstrate that radiographic CCAA are independent indicators of future adverse vascular events and that the preponde-rance of cardiovascular events rather than cerebrovascular events is consistent with recent research findings that, in the same individual, CAA is often far more advanced than CCAA (37,38). Furthermore, it is also consistent with the fact that plaque identified in the carotid bulb or internal carotid artery on B-mode ultrasound, (which is the same anatomical region imaged by panoramic radiographs) is a stronger predictor of ischemic heart disease than it is of cerebrovascular disease (39).

A likely association between diagonal ear lobe creases (DELC) and atherosclerosis of the coronary arteries (CAA) and more recently the carotid arteries has been demonstrated over the past 35 years by clinical, autopsy, and angiography studies though not completely substantiated. A series of alternative hypotheses have also been suggested, including that DELC is the result of aging and that the relationship with atherosclerotic disease is mere coincidence and even that DELC is an anatomic peculiarity of some ear lobes perhaps the result of a particular way of sleeping (40).

We, however conclude that while more research in this area is indicated, it would be prudent for dentists when conducting a head and neck cancer screening examination to observe their patients’ ears for the presence of DELC and in conjunction with the medical history, vital signs and panoramic radiograph formulate a risk assessment and determine if medical consultation is indicated for evaluation of coronary and/or carotid artery disease.
